# Association of taller stature with lower cardiovascular disease mortality in Asian people: a systematic review

**DOI:** 10.1186/s40101-019-0197-y

**Published:** 2019-06-07

**Authors:** Hiroyuki Teraura, Tatsuya Suzuki, Kazuhiko Kotani

**Affiliations:** Division of Community and Family Medicine, Center for Community Medicine, Jichi Medical University, 3311-1 Yakushiji, Shimotsuke City, Tochigi 329-0498 Japan

**Keywords:** Height, Coronary disease, Cerebrovascular disease, Stroke, Ethnicity

## Abstract

**Background:**

Adult height can serve as a disease marker. While taller stature has been reported to be linked to a decreased risk of cardiovascular disease (CVD), an influence of the height on CVD is not fully understood in specific populations of Asia, which has a lower incidence of CVD and lower stature than Western populations.

**Methods:**

We conducted a systematic review using original articles of prospective cohort studies published in English, via the PubMed database, on the relationship between the height and mortality of CVD, including cerebrovascular disease, in Asian people.

**Results:**

We selected four studies on heart/coronary disease and five studies on cerebrovascular disease. Regarding heart/coronary disease, two studies showed that taller stature was associated with a decreased mortality of heart disease in men or cardiovascular disease in women. The hazard ratios of other studies had not shown a clear significance but a decreased direction of taller stature to the mortality. Regarding cerebrovascular disease, most studies showed that taller stature was associated with a decreased mortality of total cerebrovascular diseases or stroke. In two studies, taller stature showed a decreased mortality of ischemic or hemorrhagic stroke.

**Conclusions:**

Overall, adult height may be inversely predictive to the mortality of CVD, in particular cerebrovascular disease, in Asian people. While this seems to be a similar trend to that of Westerns, further studies are warranted.

## Background

In general, human height begins increasing before adolescence and reaches its peak in early adulthood [1, 2]. Adult height is an easy indicator of physical condition and development. Stature is typically influenced by both genetic [3–6] and environmental factors. Environmental factors include nutrition [7] and socioeconomic status [8–10]. The behaviors of several hormones (i.e., insulin-like growth factor and sex hormones) are linked to genetic and environmental factors [11]. Interestingly, human height can also serve as an indicator of disease. Taller stature is linked to a decreased mortality risk, and in a meta-analysis, increases in adult height by 6.5 cm were reported to be associated with a 3% decrease in risk of death from all-cause mortality [12]. Cardiovascular disease (CVD) is a major cause of death in Western countries and Japan [13], and with every 6.5-cm increase in height, there is a 6% decrease in death from CVD (including heart/coronary and cerebral disease) [12].

Human height varies by ethnicity [14]. In Asian countries, both the mean height and the incidence of CVD (cardio- and cerebrovascular disease) are lower than in Western countries [15]. However, it is not clear whether the same relationship between the height and mortality of CVD that have been observed in Western countries is present in Asian countries. In fact, previous evaluations of the relationship between the height and mortality of CVD have been focused on Western populations. In one study, 85% of the subjects were Caucasian and it did not include a sub-analysis of racial differences [12]. Thus, we present a systematic review of the relationship between the height and mortality of CVD in Asian people. Herein, because such studies that investigate the states of CVD often include cerebrovascular disease in CVD, we considered simultaneously the mortality of cardio- and cerebrovascular disease.

## Methods

We conducted a search of the PubMed database using the terms “height,” “mortality.” and “cohort study.” The search was limited to original articles published in English up to July 5, 2018. The following search formula was used: height[All Fields] AND (“mortality”[Subheading] OR “mortality”[All Fields] OR “mortality”[MeSH Terms]) AND (“cohort studies”[MeSH Terms] OR (“cohort”[All Fields] AND “studies”[All Fields]) OR “cohort studies”[All Fields] OR (“cohort”[All Fields] AND “study”[All Fields]) OR “cohort study”[All Fields]).

**Fig. 1:**
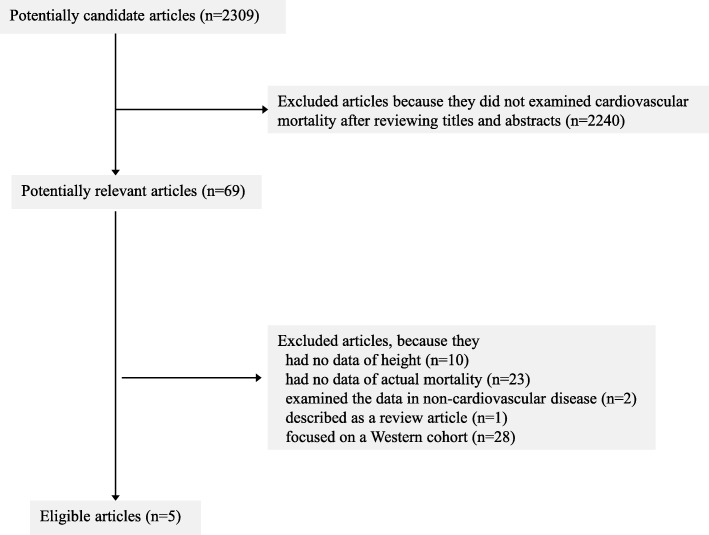
Flow of selected articles

The search returned 2309 articles, 69 of which were cohort studies that referred to both height for any age and disease mortality in the title and abstract; we then reviewed the full-text versions of those 69 articles. We excluded irrelevant articles (*n* = 64) based on lack of information on height (*n* = 10) or CVD mortality (*n* = 23) or those which were limited to studying subjects who were suffering from specific diseases (non-CVD) at the start of the study (*n* = 2). We also excluded a review article (*n* = 1) and the studies using cohorts from Western countries (*n* = 28). Five articles [16–20] that described cohort studies in Asian populations were ultimately retrieved (Fig. 1).

## Results

We retrieved five eligible studies of the relationship between the height and mortality of CVD (four studies on heart/coronary disease and five studies on cerebrovascular disease) [16–20] (Table 1). Their publication years ranged from 2003 to 2018and included studies from China (*n* = 1) [19], Japan (*n* = 2) [18, 20], and South Korea (*n* = 2) [16, 17]. The range of subjects’ mean age for the five studies was 30–74 years, the mean men’s height ranged from 155.6–173.4 cm, and the mean women’s height ranged from 144.0–160.7 cm (Fig. 1). The hazard ratios for height on mortality of CVD are shown in Table 2.

**Table 1:** Summary of the cohort studies

Ref. no.	Study name	Authors	Study subjects	Country	Sex	Mean height (cm±standard deviation)	Adjusted factors	Notes; hazard ratios for height
[16]	Korean Medical Insurance Corporation study (women)	Song and Sung, 2008	344,519 South Korean women; from1993-1994 (age, 40–64 years) for a follow-up of 10 years	South Korea	Women	155±5.2	Age, body mass index, systolic blood pressure, total cholesterol, blood glucose, smoking, alcohol, exercise, salary, occupation, residence area	Results for 5-cm increment
[17]	Korean Medical Insurance Corporation study (men)	Song et al., 2003	386,627 South Korean male civil servants; from 1992 (age, 40–64 years) for a follow-up of 6 years.	South Korea	Men	168.3±5.1	Age, body mass index, diastolic blood pressure, total cholesterol, glucose, smoking, alcohol, exercise, salary, occupation, residence area	Results for 5-cm increment
[18]	NIPPON DATA 80	Hozawa et al., 2007	3,969 and 4,955 Japanese men and women without prior cardiovascular disease; from 1980 (age, 30–92 years) for a follow-up of 19 years.	Japan	Men	162.3±6.7	Age, weight, systolic blood pressure, antihypertensive drug use, total cholesterol, diabetes mellitus, smoking, alcohol	Results for 5-cm increment
					Women	150.1±6.1		
[19]	The Shanghai Women's Health Study and the Shanghai Men's Health Study	Wang et al., 2011	61,333 Chinese men; from 2002 (age, 40–74 years, mean 54.9 years) for a follow-up of 5 years and 74,869 women from 1996 (age, 40–70 years, mean 52.1 years) for a follow-up of 5 years	China	Men	169.8	Age, birth year, body mass index, waist-hip ratio, menopause, smoking, alcohol, exercise, diet (energy, red meat, fruit and vegetables), education, income, occupation	Results for 1-standard deviation increase
					Women	157.5		
[20]	Japan Public Health Center-based Prospective Study (JPHC)	Ihira et al., 2018	50,755 and 57,039 Japanese men and women without prior cardiovascular disease; from 1990-1993 (age, 40–69 years) for a follow-up of 19.1 years(men) and 20.2 years (women).	Japan	Men	164.3±6.3	Birth year, body mass index, hypertension history, diabetes history, menopause, menarche, smoking, alcohol, exercise	Results for 5-cm increments
					Women	152.1±5.6

**Table 2:** Hazard ratios for mortality by an increase of height

Ref. no.	Sex	Deaths (n)	Hazard ratio	95% CI
Cardiovascular disease				
[19]	Men	602^a^	0.93	0.86–1.02
	Women	1018^a^	0.89	0.84–0.95*
Coronary disease				
[17]	Men	649	0.99	0.91–1.06
Heart disease				
[20]	Men	1525^b^	0.96	0.92–1.00^#^
	Women	920^b^	0.98	0.91–1.04
Ischemic heart diseases				
[16]	Women	408	0.93	0.85–1.03
[19]	Men	63	0.97	0.84–1.13
	Women	90	0.92	0.80–1.06
Myocardial infarction				
[20]	Men	579	0.99	0.92–1.06
	Women	291	0.91	0.81–1.02
Stroke				
[16]	Women	1521	0.84	0.80–0.88*
[17]	Men	1263	0.93	0.88–0.98*
[18]	Men	158^c^	0.92	0.79–1.08
	Women	168^c^	0.77	0.64–0.91*
[19]	Men	104	0.88	0.78–1.00^#^
	Women	208	0.89	0.82–0.97*
Cerebrovascular disease				
[20]	Men	1133^c^	0.95	0.90–0.99*
	Women	758^c^	0.92	0.86–0.99*
Hemorrhagic stroke				
[16]	Women	502	0.83	0.76–0.91*
[17]	Men	636	0.88	0.82–0.96*
[18]	Men	37	0.85	0.62–1.16
	Women	26	1.05	0.70–1.55
[20]	Men	512	0.89	0.82–0.96*
	Women	410	0.87	0.76–0.99*
Ischemic stroke				
[16]	Women	323	0.82	0.73–0.91*
[17]	Men	262	0.98	0.87–1.10
[18]	Men	97	0.92	0.75–1.13
	Women	69	0.66	0.51–0.84*
[20]	Men	290	1.01	0.92–1.12
	Women	185	1.02	0.89–1.17

*CI* confidence interval, *NA* not applicable

P-value: * <0.05 (significance), ^#^ approximately 0.05 (marginal significance)

^a^Includes deaths from ischemic heart disease, stroke, and other circulatory diseases

^b^Includes deaths from myocardial infarction

^c^Includes deaths from ischemic stroke, hemorrhagic stroke, and strokes of undetermined type

Regarding the results of heart/coronary disease [16, 17, 19, 20], taller stature was associated with a decreased mortality of heart disease (in men) [20] or the mortality of cardiovascular disease (this study included the diseases such as strokes) in women but not in men [19]. There were no significant associations between the height and mortality from coronary disease [17], myocardial infarction [20], or ischemic heart disease [16, 17], although all the hazard ratios showed a decreased direction (< 1.0).

Regarding the results of cerebrovascular disease [16–20], taller stature was associated with a decreased mortality of stroke [16, 17, 19] and in women but not in men [18]. Also, taller stature was associated with a decreased mortality of total cerebrovascular diseases [20], hemorrhagic stroke [16, 17, 20], or ischemic stroke (in women) [16, 18]. There were no significant associations between the height and mortality from hemorrhagic stroke [18] or ischemic stroke [17, 20], although most the hazard ratios showed a decreased direction (< 1.0).

When stratified by sex in the studies examined both sexes, taller stature was significantly associated with a decreased mortality of cardiovascular disease [19], stroke [18], and ischemic stroke [18] in only women.

## Discussion

Although there found to be limited studies on this topic, Asian populations may show a similar trend to Western populations in terms of an opposing relationship of the height with the mortality of CVD, in particular cerebrovascular disease. If this finding is further identified regardless of ethnic group, the development of height may be more noted and more investigated as a point of the prevention of CVD, besides bodily weight (this is the current mainstream).

There are several previous reports that may partly but causatively explain the inverse association of the height with the mortality of CVD. Taller statute is indicated to have vascular pathophysiological advantages. Inflammation is well-known as a major contributor to the development of CVD, and a high count of leukocyte (an inflammatory biomarker) in the blood is positively associated with taller stature [21]. While the height is affected by genetic factors [5, 6], a variation of CVD-related gene polymorphism (e.g., rs3782886), which decreases inflammation, is positively associated with taller stature [22]. Also, taller stature can induce a large diameter of coronary arteries, leading to an assumption that the large diameter predisposes to prevent the arterial occlusion [23].

Additionally, Western lifestyles (a lack of exercise with high-energy diet) are also related to an increased risk of CVD [24, 25]. The development of CVD is higher among Japanese-Americans than among Japanese people living in Japan [25]. This residence-based difference is used as an example case that lifestyle is impactful relative to genetic factors. Westernization of Asian countries may be a partial explanation on the relationship between the height and mortality of CVD in both Asian and Western populations.

Of note, in the current review, it appears that the height can have a clear influence on the mortality of cerebrovascular disease and in women. A report revealed the opposite trend among Westerns, wherein the height had a greater influence on the mortality of CVD in men than in women [26]. There are some unique gender differences regarding health outcomes among Asians. The incidence of stroke among Westerns ≥ 75 years old is higher in women than in men [27], but there is no sexual specificity with regard to the incidence of stroke in Asians [28]. In Asian countries such as Japan, cerebrovascular disease is more developed than coronary disease [15, 29]. These facts may be partly associated with the influence of height on the mortality of cerebrovascular disease and in women observed in the current review.

This review had some limitations. We acknowledged the comparatively small numbers of studies for the review and the small samples of subjects in each study. The outcomes of CVD and cerebrovascular disease were heterogeneous. More data are needed to draw more robust conclusions on this topic.

## Conclusions

From this review, overall, adult height may be inversely predictive to the mortality of CVD, in particular cerebrovascular disease, in Asian people. While this seems to be a similar trend to that of Westerns, further studies are warranted.

## Abbreviations

CVD: Cardiovascular disease

## References

[1] Sorkin JD, Muller DC, Andres R (1999). Longitudinal change in height of men and women: implications for interpretation of the body mass index. Am J Epidemiol.

[2] Perkins JM, Subramanian SV, Davey Smith G (2016). Adult height, nutrition, and population health. Nutr Rev.

[3] Silventoinen K, Pietiläinen KH, Tynelius P (2008). Genetic regulation of growth from birth to 18 years of age: the Swedish young male twins study. Am J Hum Biol.

[4] Yang J, Benyamin B, McEvoy BP (2010). Common SNPs explain a large proportion of the heritability for human height. Nat Genet.

[5] Perola M, Sammalisto S, Hiekkalinna T (2007). Combined genome scans for body stature in 6,602 European twins: evidence for common Caucasian loci. PLoS Genet.

[6] Carmichael CM, McGue M (1995). A cross-sectional examination of height, weight, and body mass index in adult twins. J Gerontol A Biol Sci Med Sci.

[7] Barker DJ, Martyn CN (1992). The maternal and fetal origins of cardiovascular disease. J Epidemiol Community Health.

[8] Kuh D, Wadsworth M (1989). Parental height: childhood environment and subsequent adult height in a national birth cohort. Int J Epidemiol.

[9] Peck MN, Lundberg O (1995). Short stature as an effect of economic and social conditions in childhood. Soc Sci Med.

[10] Kaluski DN, Chinich A, Leventhal A (2001). Overweight, stature, and socioeconomic status among women--cause or effect: Israel National Women's health interview survey, 1998. J Gend Specif Med.

[11] Gunnell D, Okasha M, Smith GD (2001). Height, leg length, and cancer risk: a systematic review. Epidemiol Rev.

[12] Emerging Risk Factors Collaboration (2012). Adult height and the risk of cause-specific death and vascular morbidity in 1 million people: individual participant meta-analysis. Int J Epidemiol.

[13] Roth GA, Johnson C, Abajobir A (2017). Global regional, and national burden of cardiovascular diseases for 10 causes, 1990 to 2015. J Am Coll Cardiol.

[14] Perkins JM, Subramanian SV, Davey Smith G (2016). Adult height, nutrition, and population health. Nutr Rev.

[15] Steg PG, Bhatt DL, Wilson PW (2007). One-year cardiovascular event rates in outpatients with atherothrombosis. JAMA.

[16] Song YM, Sung J (2008). Adult height and the risk of mortality in south Korean women. Am J Epidemiol.

[17] Song YM, Smith GD, Sung J (2003). Adult height and cause-specific mortality: a large prospective study of south Korean men. Am J Epidemiol.

[18] Hozawa A, Murakami Y, Okamura T (2007). Relation of adult height with stroke mortality in Japan: NIPPON DATA80. Stroke.

[19] Wang N, Zhang X, Xiang YB (2011). Associations of adult height and its components with mortality: a report from cohort studies of 135,000 Chinese women and men. Int J Epidemiol.

[20] Ihira H, Sawada N, Iwasaki M (2018). Adult height and all-cause and cause-specific mortality in the Japan public health center-based prospective study (JPHC). PLoS One.

[21] Shimizu Y, Yoshimine H, Nagayoshi M (2016). Short stature is an inflammatory disadvantage among middle-aged Japanese men. Environ Health Prev Med.

[22] Shimizu Y, Sato S, Noguchi Y (2017). Impact of single nucleotide polymorphism on short stature and reduced tongue pressure among community-dwelling elderly Japanese participants: a cross-sectional study. Environ Health Prev Med.

[23] Lemos PA, Ribeiro EE, Perin MA (2007). Angiographic segment size in patients referred for coronary intervention is influenced by constitutional, anatomical, and clinical features. Int J Cardiovasc Imaging.

[24] Shigetake S (2008). Heart disease in Asia. Circulation.

[25] Sekikawa A, Curb JD, Ueshima H (2008). Marine-derived n-3 fatty acids and atherosclerosis in Japanese, Japanese-American, and white men: a cross-sectional study. J Am Coll Cardiol.

[26] Batty GD, Shipley MJ, Gunnell D (2009). Height, wealth, and health: an overview with new data from three longitudinal studies. Econ Hum Biol.

[27] Hart RG, Pearce LA, McBride R (1999). Factors associated with ischemic stroke during aspirin therapy in atrial fibrillation: analysis of 2012 participants in the SPAF I-III clinical trials. The stroke prevention in atrial fibrillation (SPAF) investigators. Stroke.

[28] Inoue H, Atarashi H, Okumura K (2014). Impact of gender on the prognosis of patients with nonvalvular atrial fibrillation. Am J Cardiol.

[29] Ueshima H (2007). Explanation for the Japanese paradox: prevention of increase in coronary heart disease and reduction in stroke. J Atheroscler Thromb.

